# Progesterone Modulates Placental Expression of p53 and β-Catenin in a Zone-Specific Manner in Dexamethasone-Treated Rats

**DOI:** 10.7759/cureus.90457

**Published:** 2025-08-19

**Authors:** Ali Alkhabbaz, Abdullah Alnaqeeb, Fahad Alabduljader, Yousef AlSalem, Maryam A Alqaryan, Mariam M Alawadhi, Maie D Al-Bader

**Affiliations:** 1 Medicine, Al-Amiri Hospital, Kuwait City, KWT; 2 General Surgery, Jaber Al-Ahmed Jaber Al-Sabah Hospital, Kuwait City, KWT; 3 Orthopedic Surgery, Jaber Al-Ahmed Jaber Al-Sabah Hospital, Kuwait City, KWT; 4 Orthopedics, Al-Razi Hospital, Kuwait city, KWT; 5 Physiology, Health Sciences Center, Kuwait City, KWT; 6 Physiology, Health Sciences Center, Kuwait, KWT

**Keywords:** basal zone, intrauterine growth restriction, labyrinth zone, p53, progesterone, β-catenin

## Abstract

Introduction

The placenta is a transient pregnancy organ, and its development depends on proliferation and apoptosis throughout pregnancy. Abnormal placental development can result in intrauterine growth restriction (IUGR). Progesterone has a major role in regulating cell death and survival in various tissues, and its low maternal levels are highly correlated with IUGR.

Aim

The aim of this article was to evaluate the modulatory effect of progesterone on placental cell death and survival in a rat model of dexamethasone-induced IUGR.

Methods

Pregnant Sprague-Dawley rats were treated with a daily intraperitoneal (i.p.) injection: control (saline), dexamethasone, dexamethasone and progesterone, and progesterone. Injections started from 15 days of gestation (dg) until the end of the experiment at 21 days of gestation. The fetus and the placenta were weighed, and the placental labyrinth and basal zones were weighed and taken for gene and protein analysis of p53 and β-catenin.

Key results

Dexamethasone treatment resulted in decreased maternal progesterone levels, and fetal body and placental basal and labyrinth zone weights. Dexamethasone treatment significantly increased p53 expression in the basal and labyrinth zones and decreased β-catenin expression in the basal zone only. Progesterone co-supplementation with dexamethasone alleviated the increase in p53 levels in the labyrinth zone only and restored the expression of β-catenin in the basal zone.

Conclusion

Despite progesterone modulation of cell survival and growth, the effect was not translated into improved fetal or placental weights. Implication: These results highlight the importance of the zone-specific effect of progesterone in modulating cell death and survival in the placental complications.

## Introduction

The placenta is a transient organ that develops during pregnancy. The rat placenta is a chorioallantoic placenta with trophoblast stem cells differentiated into the basal zone (BZ) and the labyrinth zone (LZ). The basal zone is the site of placental hormone production, while the labyrinth zone serves as a maternal-fetal barrier for nutrient and waste exchange [1]. Apoptosis is a normal process during placental development, especially during mid-late gestation, but increased placental apoptosis is associated with intrauterine growth restriction (IUGR) [2-4]. Dexamethasone-induced IUGR placentas show a higher apoptotic rate and alterations in the expression of placental cell cycle control proteins, including p53. The tumor suppressor protein p53 regulates the cell cycle checkpoint in response to DNA damage and triggers apoptosis when the damage is irreparable. Dexamethasone treatment in pregnant rats increased placental DNA fragmentation in a zone-specific manner and increased p53 expression, and this effect was associated with reduced placental and fetal body weights [4, 5]. The Wnt/β-catenin signaling pathway plays a prominent role in maintaining cellular homeostasis. β-catenin is the key protein of the Wnt/β-catenin pathway, regulating the expression of multiple genes that influence various cellular functions, including cell proliferation and differentiation, apoptosis, and inflammation-associated cancer in humans [6]. β-catenin plays a crucial role during vertebrae placentation, as it is involved in trophoblast invasion, angiogenesis, and embryonic development [7].

Progesterone is essential for maintaining pregnancy, with its levels increasing progressively throughout gestation, dropping before labor. Progesterone increases rat placental angiogenesis and has a vasorelaxant effect on fetoplacental vasculature [8, 9]. Low maternal progesterone levels were associated with placental insufficiency, reduction in fetal body weight, and development of IUGR in humans [10-12]. Several studies confirmed progesterone's protective role against apoptosis in various tissues [13, 14]. Based on the known role of progesterone in proliferation and apoptosis control, this study aims to evaluate progesterone's modulatory effect on apoptosis in the placental basal and labyrinth zones by examining the expression of p53 and β-catenin. The study also aims to assess whether progesterone treatment improves placental weight and prevents the development of IUGR in dexamethasone-treated rats.

## Materials and methods

Experiment design

**Figure 1:**
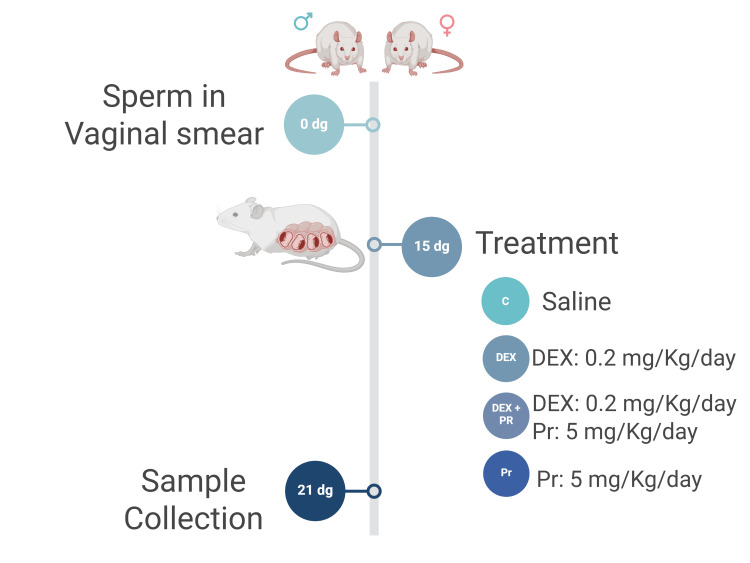
Experiment design

Adult Sprague-Dawley rats were maintained under standard conditions and diet at the Animal Resources Centre at the Health Sciences Center, Kuwait University. All experiments were conducted per the National Institutes of Health (NIH) and Animal Research guidelines for laboratory animal care at Kuwait University, approved in December 2020. Following a pilot study to standardize the model, the main experiment and sample collection were conducted in 2022. The experimental design was explained previously [2]. Briefly, pregnant rats (n=6/group) were randomly divided into four groups based on the treatment regimen: control group (C: saline), dexamethasone-treated group (DEX: 0.2 mg/Kg/day), dexamethasone and progesterone-treated group (DEX+Pr: DEX-0.2 mg/Kg/day + Pr-5 mg/Kg/day) and progesterone-treated group (Pr: 5 mg/Kg/day). Rats received daily intraperitoneal (i.p.) injections from 15 days of gestation (dg) to 20 dg between 8:30 and 10:30 am. The animals were sacrificed at the end of gestation (21 dg) when the fetal growth reaches its maximum potential [1, 15]. The progesterone dose was adopted from a previous study where comparative doses of progesterone were used to prevent preterm birth [16]. Both dexamethasone and progesterone were saline-soluble and purchased from Sigma-Aldrich, Germany. The experiment design is shown in Figure 1.

Tissue collection

The experiments were terminated at 21 dg, as described previously [17]. The blood was collected from the left ventricle of the heart, and fetuses were cleaned and then weighed. Placental basal and labyrinth zones were separated using forceps, as the boundaries of the zones are clearly defined. Each zone was then weighed, pooled, and stored at -70°C until use. Samples used for ReT-PCR were immersed in liquid nitrogen, while the cryoprotective agent dimethylsulfoxide (DMSO, 10% v/v, Sigma-Aldrich, Germany) was added to samples used for Western blotting analysis before storage. Only pregnancies with a conceptus range between six and 13 weeks were included in this study, as the number of concepts out of this range has a significant inverse relationship with the fetal weight [15].

Measurement of progesterone level

Maternal serum progesterone level was measured using a rat progesterone enzyme-linked immunosorbent assay (ELISA) (Catalogue no. E-EL-0154, Elabscience, Texas, United States). The kit has a detection range of 0.31-20ng/ml and a sensitivity of 0.15ng/ml. The procedure was conducted according to the manufacturer's instructions.

Real-time polymerase chain reaction analysis

Gene expressions of Tp53 and β-catenin (CTNN1) in placental basal and labyrinth zones were studied using Real-time polymerase chain reaction (ReT-PCR) as described previously [5, 18]. Briefly, TRIzol method was used to extract the total RNA (Invitrogen Corporation, Massachusetts, USA), and then the total RNA was estimated by spectrophotometry. The purity and integrity of RNA were confirmed by agarose gel electrophoresis stained with ethidium bromide. Only pure and integrated RNA was used. The RNA samples were DNase-treated before reverse transcription as described previously [18]. The ReT-PCR reaction was carried out in a ReT-PCR system (model 7500; Applied Biosystems, California, USA). The ReT-PCR reactions were performed using primer sets and an endogenous control, as specified in Table 1. All primer sets were purchased from ThermoFisher Scientific, USA TaqMan Universal Master Mix (Catalogue no. 4369016; Applied Biosystems, USA) was used to prepare the PCR reactions following the previously described protocol [5]. All other materials used in ReT-PCR experiments were purchased either from Invitrogen Corporation, USA, or Applied Biosystems, USA polymerase chain reaction cycling conditions were as follows: an initial step at 50°C for two minutes (one cycle), followed by 95°C for 10 minutes (one cycle), then 40 cycles at 95°C for 15 seconds, and finally 60°C for one minute over 60 cycles. Relative quantification was employed to assess changes in target gene expression compared to the calibrator, which consisted of control placentas collected at 21 dg.

**Table 1:** Primer specifications

Primer	Catalogue no.	Primer sequence
Tp53	4331182	Forward sequence: CCTCAGCATCTTATCCGAGTGG, Reverse sequence: TGGATGGTGGTACAGTCAGAGC
CTNNB1	4351372	Forward sequence: CACAAGCAGAGTGCTGAAGGTG, Reverse sequence: GATTCCTGAGAGTCCAAAGACAG
ACTB	4352340E	Forward sequence: CCCGCGAGTACAACCTTCT, Reverse sequence: CGTCATCCATGGCGAACT

Western blotting

The protein expression of p53 and β-catenin was evaluated as described previously [2]. Briefly, tissues were homogenized, and the protein content was measured by Bradford assay [19, 20]. The samples were prepared for loading as explained in our earlier work [17]. After separating the protein in precast gels (Mini-Protean TGX Stain Free Gel 4-20%, Bio-RAD, California, USA), the proteins were transferred to methanol-activated polyvinylidene difluoride (PVDF) membranes. Membranes were then stained with Ponceau red stain (0.1% w/v Ponceau S, 5% v/v acetic acid) to evaluate the total protein optical density (OD) using the Bio-RAD ChemiDOCTM MP imaging system. Ponceau stain is approved to be used as a loading control [21]. After washing, the membranes were blocked (1x TBS 1% Casein Blocker, Bio-RAD, California, USA). Then, membranes were incubated overnight at 4°C with the primary antibody, as specified in Table 2. The next morning, the membranes were washed and incubated with the appropriate secondary antibody (Table 2), followed by incubation with Clarity Western ECL Substrate (Catalogue no. 1705060, Bio-RAD, California, USA). Densitometric analysis was carried out for all protein bands using the Bio-RAD ChemiDOCTM MP Imaging system.

**Table 2:** Antibody specifications and concentrations

Concentration	Company	Catalog number	Secondary antibody	Concentration	Company	Species and clone	Catalog number	Primary antibody
1:1000	Invitrogen	31570	Alex Flour 555 donkey anti-mouse IgG	1:200	Santa Cruz Biotechnology	Mouse monoclonal	sc-99	p53 (Pab240)
1:10,000	Santa Cruz Biotechnology	sc-516102	m-IgGk BP-HRP	1:500	Santa Cruz Biotechnology	Mouse monoclonal	sc-597373	β-catenin (12F7)

Statistical analysis

ReT-PCR results were calculated using a comparative method (described by Livak et al. [22]). The ∆Ct value was determined by subtracting the average Ct value of the housekeeping gene ACTB from the average target CT value of each of the target genes (Tp53 and CTNNB1). The ∆∆Ct was determined by subtracting the ∆Ct of the calibrator (control group at 21dg) from that of each respective sample. The normalized expression was determined by using 2-∆∆Ct. For protein studies, the OD value of each protein (p53 and β-catenin) was divided by the OD of the total protein evaluated by the Ponceau stain. The statistically significant difference in the data was evaluated using SPSS (Software Package for Statistical Analysis, version 26; IBM Inc., Armonk, New York). ANOVA test was used, followed by least significant difference (LSD) post-hoc analysis when the test for homogeneity of variances was fulfilled, or Games-Howell post-hoc analysis when the homogeneity of variances was not attained. Values are represented as mean ± standard error of the mean (SEM), and p<0.05 was considered the minimum level of significance.

## Results

Progesterone treatment did not alleviate the decrease in maternal progesterone levels, nor did it prevent IUGR

**Figure 2:**
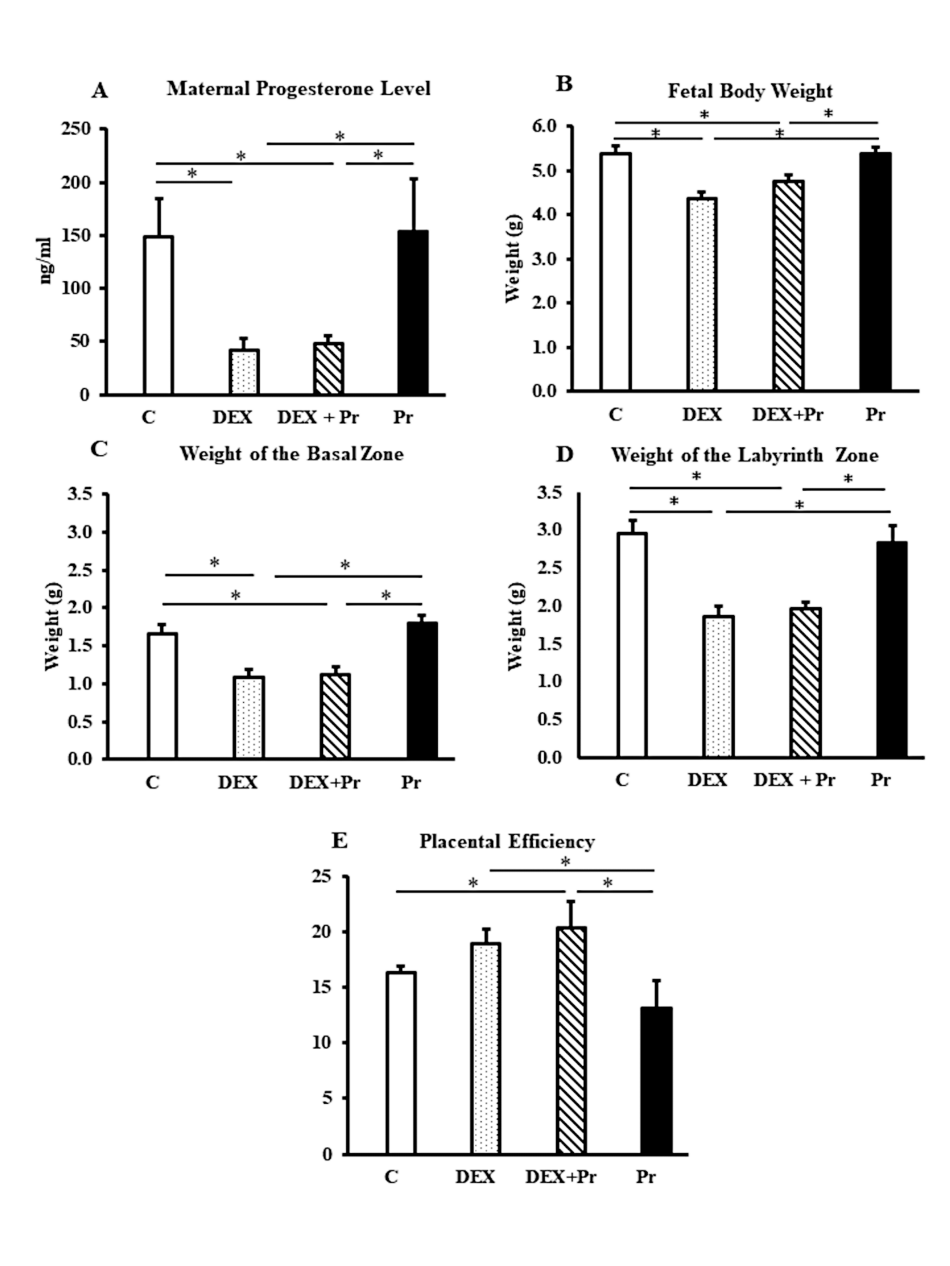
Animal gross data Maternal progesterone level (A), fetal body weights (B), basal zone weights (C), labyrinth zone weights (D) and placental efficiency (E) in control (C), dexamethasone (DEX), dexamethasone + progesterone (DEX+Pr) and progesterone (Pr) groups (n=6). Data represent mean + standard error of the mean (SEM). *p<0.05

Maternal progesterone levels decreased significantly in both DEX and DEX+Pr compared to the control group (*p<0.05; Figure 2A). Co-treatment of dexamethasone and progesterone did not prevent the reduction in maternal progesterone levels caused by dexamethasone. The progesterone group is used as a positive control, and there was no significant difference between the control and progesterone groups in terms of maternal progesterone levels (Figure 2A).

Dexamethasone treatment induced IUGR and decreased fetal body weight and basal and labyrinth zone weights compared to both control groups (*p<0.05; Figure 2B-D). Progesterone co-treatment with dexamethasone did not prevent the reduction in any of the weights measured. Placental efficiency is a calculated measure that evaluates fetal development per placental development (fetal body weight/placental weight). The placental efficiency significantly increased in the DEX+Pr-treated group compared to both control groups (C and Pr) (*p<0.05; Figure 2E).

Progesterone treatment prevented the increase in p53 expression induced by dexamethasone treatment in the placental labyrinth zones

**Figure 3:**
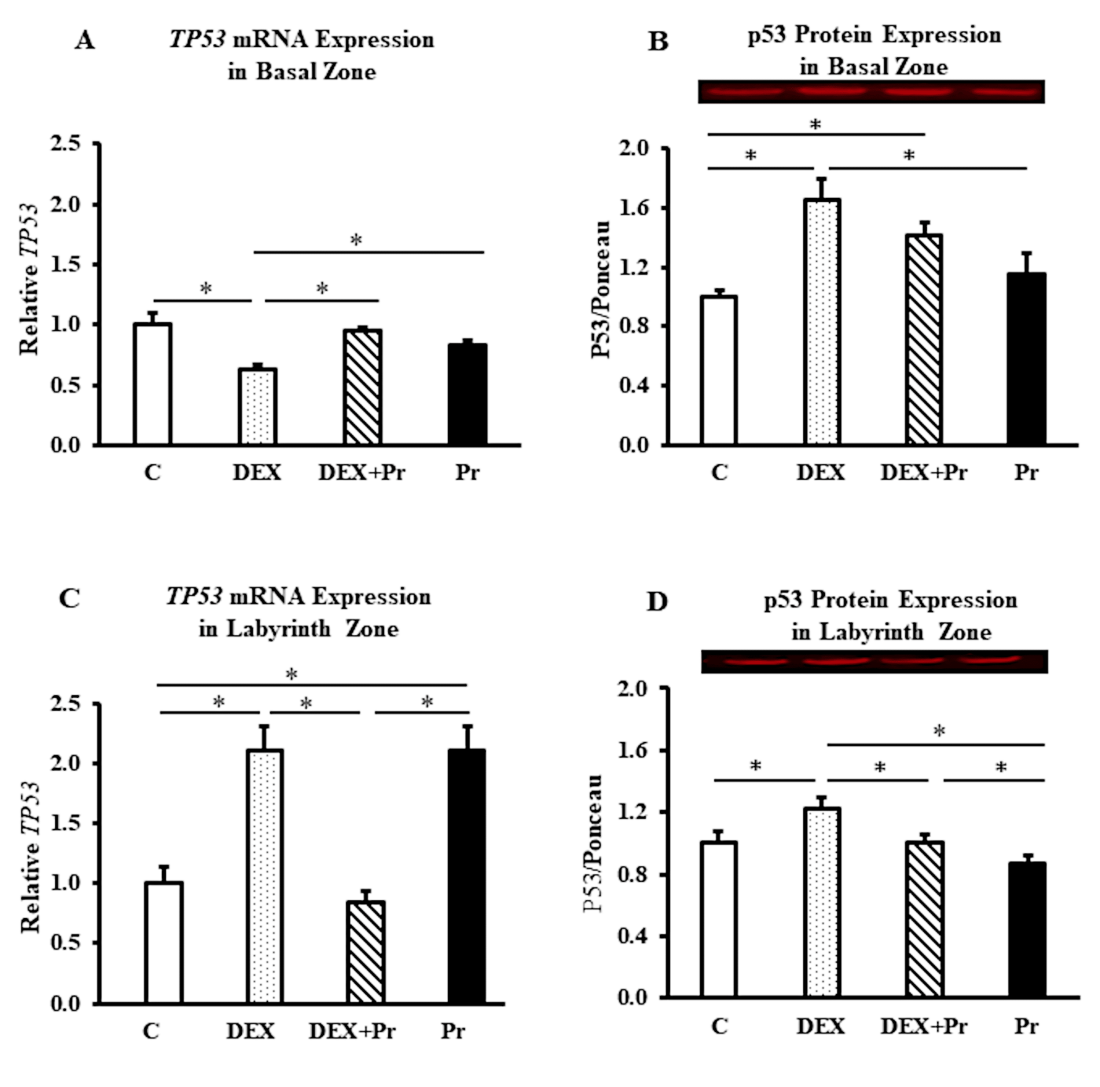
p53 mRNA and protein expression in the basal and labyrinth zones The basal zone expression of Tp53 mRNA (A) and p53 protein (B), the labyrinth zone expression of Tp53 mRNA (C) and p53 protein (D) in control (C), dexamethasone (DEX), dexamethasone + progesterone (DEX+Pr) and progesterone (Pr) groups (n=6). Data represent mean + standard error of the mean (SEM). *p<0.05

In the basal zone, the mRNA expression of the Tp53 gene was significantly reduced in the dexamethasone-treated group compared to the control group, and progesterone co-treatment prevented this reduction (*p<0.05; Figure 3A). However, at the protein level, p53 expression increased significantly in both dexamethasone-treated groups (DEX and DEX+Pr) compared to the control group (*p<0.05; Figure 3B).

In the labyrinth zone, the mRNA expressions of p53 increased significantly in the DEX-treated group compared to the control and DEX+Pr groups (gene), and to all other groups (protein) (*p<0.05; Figure 3C-D). Progesterone co-treatment with dexamethasone (DEX+Pr group) decreased p53 mRNA and protein expressions significantly compared to the DEX group (*p<0.05; Figure 3C-D).

Progesterone modulation of β-catenin expression at the gene and protein levels varies in different placental zones

**Figure 4:**
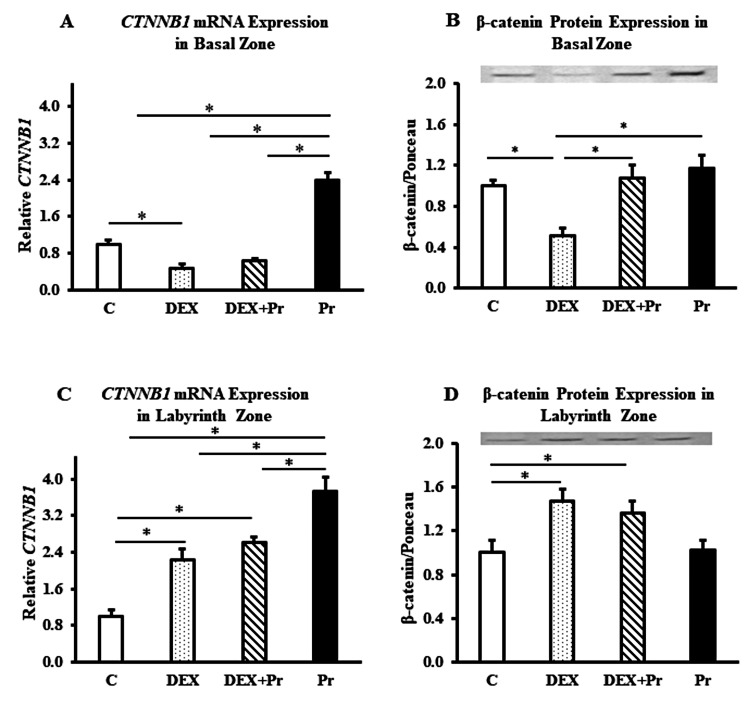
β-catenin mRNA and protein expression in the basal and labyrinth zones The basal zone expression of CTNNB1 mRNA (A) and β-catenin protein (B), the labyrinth zone expression of CTNNB1 mRNA (C) and β-catenin protein (D) in control (C), dexamethasone (DEX), dexamethasone + progesterone (DEX+Pr) and progesterone (Pr) groups (n=6). Data represent mean + standard error of the mean (SEM). *p<0.05

In the basal zone, both mRNA and protein expressions of β-catenin were reduced significantly in the dexamethasone-treated group compared to the control group (*p<0.05; Figure 4A-B). Progesterone co-treatment with dexamethasone significantly increased the protein expression of β-catenin compared to the DEX group (*p<0.05; Figure 4B).

In the labyrinth zone, the gene and protein expressions of β-catenin increased significantly in both DEX-treated groups (DEX and DEX+Pr) compared to the control group (*p<0.05; Figure 4C-D). Progesterone co-treatment did not affect the gene or protein expression of β-catenin compared to the dexamethasone-treated group, as it remained significantly higher than in the control group (Figure 4C-D).

## Discussion

Progesterone treatment increased placental efficiency but did not prevent IUGR

Towards the end of pregnancy, the basal zone undergoes apoptosis, indicating a reduction in the functional importance of this zone in hormone production [4]. Dexamethasone exaggerates the apoptotic process in the basal zone [4], which is translated as smaller basal zones seen in the present study and earlier studies [2, 17]. The effect of dexamethasone on exaggerating placental apoptosis was associated with a reduction in the levels of human placental lactogens that stimulate ovarian progesterone production [23], leading to lower maternal progesterone levels. In addition, dexamethasone upregulates the expression of cytochrome P450 that hydroxylates progesterone to its metabolites in human adipose tissue [24]. These changes account for the lower maternal progesterone levels detected in the dexamethasone-treated groups in the present study. The increase in progesterone metabolism caused by dexamethasone is believed to prevent the increase in maternal progesterone levels when progesterone was supplemented in the DEX+Pr group.

Dexamethasone treatment in pregnant rats induces alterations in the expression of cell cycle control proteins, including p53. The significant upregulation of placental p53 expression in IUGR pregnancies was associated with smaller basal and labyrinth zones. The labyrinth zone is responsible for fetal-maternal nutrient exchange and contributes directly to fetal growth. So, smaller fetuses seen in our study are a direct result of smaller labyrinth zones due to apoptosis induced by dexamethasone treatment.

The increase in placental efficiency is believed to be a compensatory mechanism to maintain fetal growth at the expense of placental development. This is supported by a higher reduction in placental weight compared to fetal body weight in DEX and DEX+Pr groups (fetal body weight reduced by 18.9% and 11.8%, and placental weight reduced by 30% and 23%, respectively). Despite the increased placental efficiency with progesterone co-treatment, this was not translated at the level of improved fetal or placental weights, and it did not recover IUGR.

Progesterone modulatory effect on apoptosis and cell survival is zone-specific

Interestingly, progesterone co-treatment with dexamethasone prevented the upregulation of p53 expression in the placental labyrinth zones, despite the significant reduction in maternal progesterone level measured in the DEX+Pr group compared to the control group. Our previous study demonstrated that dexamethasone treatment downregulated nuclear and membrane progesterone receptors in rat placentas. However, progesterone co-treatment with dexamethasone significantly upregulated the expression of placental progesterone receptors [9]. The upregulation of progesterone receptors indicates an increase in placental sensitivity to progesterone. This could explain the progesterone effect on decreasing p53 expression with progesterone co-treatment, despite the unchanged progesterone level observed when progesterone was supplemented. These findings suggest a direct protective effect of progesterone against apoptosis by decreasing placental p53 expression.

The protein β-catenin activates the Wnt signaling pathway, inducing proliferation and differentiation. The response of β-catenin to dexamethasone is different in different placental zones. In the labyrinth zone, β-catenin expression increased with dexamethasone treatment. This is believed to be a protective mechanism against apoptosis due to the continuous proliferation occurring in the labyrinth zone throughout gestation in the rat placenta [1, 25]. On the contrary, the basal zone expression of β-catenin decreased significantly with dexamethasone treatment. This indicates that the higher apoptotic rate, supported by higher p53 levels detected, was less opposed by increased proliferation and resulted in a smaller basal zone. The difference in progesterone effect on β-catenin in basal and labyrinth zones is believed to be due to dissimilar expression of progesterone receptors in each of these zones [9, 26]. Besides the higher expression of nuclear progesterone receptors in the basal zone, the expression of membrane progesterone receptors, alpha and beta, is much higher in the basal zone and extremely low in the labyrinth zone at late gestation in rats [27]. This demonstrates the role of zone-specific expression of progesterone receptors in modulating the effect of progesterone on proliferation. However, progesterone's modulatory effect on the expression of cell death and survival genes and proteins was not translated into improved fetal or placental weights.

A key limitation of this study is the timing of sample collection. The selected time point (21 dg) represents the end of gestation, when fetal growth reached its maximum potential. However, including earlier time points, when the placenta growth peaks (19 dg), would provide a more comprehensive understanding of progesterone's effects on the placenta. Unfortunately, this was not feasible due to constraints in time and resources.

## Conclusions

The present study emphasizes the role of progesterone in preventing the increase in pro-apoptotic marker p53 and upregulating anti-apoptotic marker β-catenin in different placental zones of dexamethasone-induced IUGR. Despite the significant improvement of placental efficiency when progesterone was co-treated with dexamethasone, progesterone supplementation failed to maintain fetal or placental weights. This finding can suggest a future study evaluating progesterone-dose-dependent effects on improving fetal and placental weight in IUGR pregnancies.

## References

[1] Furukawa S, Tsuji N, Sugiyama A (2019). Morphology and physiology of rat placenta for toxicological evaluation. J Toxicol Pathol.

[2] Alawadhi M, Mouihate A, Kilarkaje N, Al-Bader M (2022). Progesterone partially recovers placental glucose transporters in dexamethasone-induced intrauterine growth restriction. Reprod Biomed Online.

[3] Ain R, Canham LN, Soares MJ (2005). Dexamethasone-induced intrauterine growth restriction impacts the placental prolactin family, insulin-like growth factor-II and the Akt signaling pathway. J Endocrinol.

[4] Waddell BJ, Hisheh S, Dharmarajan AM, Burton PJ (2000). Apoptosis in rat placenta is zone-dependent and stimulated by glucocorticoids. Biol Reprod.

[5] Alqaryyan M, Kilarkaje N, Mouihate A, Al-Bader MD (2017). Dexamethasone-induced intrauterine growth restriction is associated with altered expressions of metastasis tumor antigens and cell cycle control proteins in rat placentas. Reprod Sci.

[6] MacDonald BT, Tamai K, He X (2009). Wnt/beta-catenin signaling: components, mechanisms, and diseases. Dev Cell.

[7] Cadigan KM, Nusse R (1997). Wnt signaling: a common theme in animal development. Genes Dev.

[8] Paonessa DJ, Shields AD, Howard BC, Gotkin JL, Deering SH, Hoeldtke NJ, Napolitano PG (2006). 17-Hydroxyprogesterone caproate reverses induced vasoconstriction of the fetoplacental arteries by the thromboxane mimetic U46619. Am J Obstet Gynecol.

[9] Alawadhi M, Kilarkaje N, Mouihate A, Al-Bader MD (2023). Role of progesterone on dexamethasone-induced alterations in placental vascularization and progesterone receptors in rats†. Biol Reprod.

[10] Yanaihara T, Hirato K, Seo F (1984). Prenatal diagnosis of IUGR by assessing the multiple hormone concentration in maternal peripheral blood (Article in Japanese). Nihon Sanka Fujinka Gakkai Zasshi.

[11] Bartholomeusz RK, Bruce NW, Lynch AM (1999). Embryo survival, and fetal and placental growth following elevation of maternal estradiol blood concentrations in the rat. Biol Reprod.

[12] Hay WW Jr, Brown LD, Rozance PJ, Wesolowski SR, Limesand SW (2016). Challenges in nourishing the intrauterine growth-restricted foetus - lessons learned from studies in the intrauterine growth-restricted foetal sheep. Acta Paediatr.

[13] Morrissy S, Xu B, Aguilar D, Zhang J, Chen QM (2010). Inhibition of apoptosis by progesterone in cardiomyocytes. Aging Cell.

[14] Wang Y, Abrahams VM, Luo G, Norwitz NG, Snegovskikh VV, Ng SW, Norwitz ER (2018). Progesterone inhibits apoptosis in fetal membranes by altering expression of both pro- and antiapoptotic proteins. Reprod Sci.

[15] Furukawa S, Hayashi S, Usuda K, Abe M, Hagio S, Ogawa I (2011). Toxicological pathology in the rat placenta. J Toxicol Pathol.

[16] Hashimoto H, Eto T, Endo K, Itai G, Kamisako T, Suemizu H, Ito M (2010). Comparative study of doses of exogenous progesterone administration needed to delay parturition in Jcl:MCH(ICR) mice. Exp Anim.

[17] Alawadhi MM, Al Shammari F, Ali FM, Almatar R, Al-Duwaikhi A, Al-Bader MD (2022). The effect of progesterone administration on the expression of metastasis tumor antigens (MTA1 and MTA3) in placentas of normal and dexamethasone-treated rats. Mol Biol Rep.

[18] Al-Bader MD, Al-Sarraf HA (2005). Housekeeping gene expression during fetal brain development in the rat-validation by semi-quantitative RT-PCR. Brain Res Dev Brain Res.

[19] Bradford MM (1976). A rapid and sensitive method for the quantitation of microgram quantities of protein utilizing the principle of protein-dye binding. Anal Biochem.

[20] Al-Bader MD, Kilarkaje N, El-Farra A, Al-Abdallah AA (2015). Expression and subcellular localization of metastasis-associated protein 1, its short form, and estrogen receptors in rat placenta. Reprod Sci.

[21] Romero-Calvo I, Ocón B, Martínez-Moya P, Suárez MD, Zarzuelo A, Martínez-Augustin O, de Medina FS (2010). Reversible Ponceau staining as a loading control alternative to actin in Western blots. Anal Biochem.

[22] Livak KJ, Schmittgen TD (2001). Analysis of relative gene expression data using real-time quantitative PCR and the 2(-Delta Delta C(T)) Method. Methods.

[23] Lange AP, Anthonsen H (1980). Serum levels of human placental lactogen during and after prenatal dexamethasone therapy. Acta Obstet Gynecol Scand.

[24] Khalil MW, Strutt B, Vachon D (1994). Effect of dexamethasone and cytochrome P450 inhibitors on the formation of 7 alpha-hydroxydehydroepiandrosterone by human adipose stromal cells. J Steroid Biochem Mol Biol.

[25] Rudge MV, Costa E, Barbisan LF (2012). Evaluation of cell proliferation and apoptosis in placentas of rats with severe diabetes. Brazilian Archives of Biology and Technology.

[26] Ain R, Konno T, Canham LN, Soares MJ (2006). Phenotypic analysis of the rat placenta. Methods Mol Med.

[27] Mark PJ, Smith JT, Waddell BJ (2006). Placental and fetal growth retardation following partial progesterone withdrawal in rat pregnancy. Placenta.

